# Guillain–Barré syndrome presenting with Raynaud’s phenomenon: a case report

**DOI:** 10.1186/s12883-014-0174-3

**Published:** 2014-09-03

**Authors:** Sonali Sihindi Chapa Gunatilake, Harith Wimalaratna

**Affiliations:** 1 Teaching Hospital, Kandy, Sri Lanka

**Keywords:** Guillain–Barré syndrome, Raynaud’s phenomenon, Acute inflammatory demyelinating polyneuropathy, Cerebrospinal fluid, Cyto-protein dissociation, Acute ascending flaccid paralysis

## Abstract

**Background:**

Guillain–Barré syndrome is an immune mediated acute inflammatory polyradiculo-neuropathy involving the peripheral nervous system. Commonest presentation is acute or subacute flaccid ascending paralysis of limbs. Rarely autonomic dysfunction can be the presenting feature of Guillain–Barré syndrome. Raynaud’s phenomenon, although had been described in relation to many disease conditions, has not been described in association with Guillain–Barré syndrome up to date.

**Case presentation:**

We report the first case of Guillain–Barré syndrome presenting with Raynaud’s phenomenon in a 21-year-old previously well boy. New onset Raynaud’s phenomenon was experienced followed by acute ascending flaccid paralysis of lower limbs and upper limbs together with palpitations and postural giddiness. Nerve conduction studies showed acute inflammatory demyelinating polyneuropathy with cerebrospinal fluid cyto-protein dissociation. He was treated with intravenous immunoglobulin and showed a satisfactory clinical recovery of muscle weakness, Raynaud’s phenomenon and autonomic disturbances.

**Conclusion:**

Guillain–Barré syndrome presenting with Raynaud’s phenomenon is not being reported in literature previously. Although the underlying mechanism is not fully understood, Raynaud’s phenomenon should prompt the physician to consider Guillain–Barré syndrome with a complimentary clinical picture.

## Background

Guillain–Barré syndrome is an acute inflammatory polyradiculo-neuropathy, an immune mediated disease affecting the peripheral nervous system. It comprises of a heterogeneous group of clinical entities. Usually it is preceded by an antecedent event in two thirds of the patients, commonly an infection [1],[2]. Typical presentation is of acute ascending flaccid paralysis of lower-limbs with absent or diminished reflexes, extending more proximally with time and proximal muscle groups being affected more than the distal. Many patients experience paresthesia with mild degree of sensory involvement. Cranial nerve involvement is also not uncommon. Dysautonomia had been described in 60% of the patients with Guillain–Barré syndrome [3] manifesting with reduced or excess activation of sympathetic or parasympathetic nervous systems. Rarely autonomic involvement can be the presenting symptom in Guillain–Barré syndrome [2],[4],[5].

Raynaud’s phenomenon is an exaggerated vasospastic response to emotion or cold weather giving rise to tri-phasic color change in the acral regions of the body [5],[6]. Classic color changes are white (due to ischaemia from vasospasms), blue (due to deoxygenation of blood) and red (due to reperfusion). Variety of causes and associated diseases have been described to give rise to Raynaud’s phenomenon but an extensive literature survey did not reveal Raynaud’s phenomenon in association with Guillain–Barré syndrome. Herein, we report the first case of Guillain–Barré syndrome manifesting with Raynaud’s phenomenon as the presenting complaint.

## Case presentation

A 21-year-old previously healthy male presented to Teaching Hospital, Kandy, Sri Lanka in July 2013 with the complaint of bluish discoloration of hands for 5 days duration and subsequent weakness of limbs.

He had experienced repeated transient episodes of hand discoloration, blanching followed by bluish discoloration. There was similar involvement of the feet. Episodes occurred several times per day with increasing in frequency. It was not associated with pain, numbness or weakness of the limbs, neck pain or radicular pain. Identified precipitant was cold environment, but was not associated with emotional stress. There was no history of trauma. He did not have recent weight loss, anorexia, fever, oral ulcers, hair-loss, skin rashes, joint swelling or joint pain. No similar episodes were observed in the past.

Five days after the onset of hand discoloration, he noticed weakness of both lower limbs with difficulty in walking which progressed to weakness of upper limbs, predominantly the proximal parts. He was able to walk with support without ataxia initially and his hand grip was poor and dropped certain objects. The weakness was progressive. There was no double vision, slurring of speech, dysphagia or dyspnea. There was neither bladder nor bowel involvement. He had tingling sensation of fingers and toes at the onset of the weakness of limbs but remained static. He did not have postural giddiness but experienced intermittent palpitations. He denied excessive sweating or hypersalivation. Raynaud’s phenomenon which he experienced earlier was still present during this period of illness. There was no preceding respiratory tract illness or diarrhoeal illness. His past medical history was not significant and there was no exposure to heavy metals. He did not have any foreign travels or exposure to tick bites. The patient denied any long term use of medication and he was a non-smoker and did not consume alcohol. He was unmarried & denied any sexual misbehavior.

On examination, he was otherwise well looking male with a body mass index of 21 kg/m^2^. Generalized weakness of muscle groups were noted involving distal more than the proximal in both upper and lower limbs (upper limb: distal - grade 3/5, proximal – grade 4/5; lower limb: distal – grade 2/5, proximal – 3/5). There was generalized areflexia with hypotonia. Pupils were size 3, equally reacting to light. Sensory system, cerebellar system, cranial nerves and fundi were clinically normal. Raynaud’s phenomenon was observed when hands were exposed to cold. There were no digital ulcers or gangrene. Cardiovascular system examination revealed a regular heart rate of 110/min with a postural drop in systolic blood pressure (supine – 130/80; seated – 100/80). All peripheral pulses were present with good volume and there were no subclavian, carotid or femoral bruits. Respiratory, abdominal and locomotor system examinations were normal.

His complete blood count, blood picture, renal and liver profile were normal. Other basic investigations revealed; erythrocyte sedimentation rate – 05 mm in 1st Hour, C-reactive protein – normal, serum electrolytes including Na^+^, K^+^, Ca^2+^ & Mg^2+^ were normal, serum proteins: total– 7.8 g/dl, albumin - 4.6 g/dl, globulin - 3.1 g/dl. Fasting blood sugar and lipid profile were within normal range. Cerebrospinal fluid (CSF) studies done on day 10 of the illness showed elevated proteins (120 mg/dl; normal < 40 mg/dl) with 01 white cell, 100% being lymphocytes. CSF sugar was within normal limits compared to plasma value. Nerve conduction studies revealed focal segmental demyelinating type of neuropathy with elevated distal motor latency, elongated F-wave, conduction block without axonal abnormalities, concluding as acute inflammatory demyelinating polyneuropathy (AIDP) type Guillain–Barré syndrome.

Further investigations showed negative serology for mycoplasma, campylobacter jejuni, cytomegalovirus, Epstein-Barr virus, hepatitis B & C and human immuno deficiency virus (HIV). Anti-nuclear antibodies, anti-neutrophil antibodies and rheumatoid factor were negative with normal thyroid stimulating hormone levels. Serum protein electrophoresis was normal. X ray cervical spine, thoracic inlet and chest x ray were negative for cervical ribs or any significant pathology. Venous duplex scan of the upper limbs showed good arterial flow without obstruction. Electrocardiogram showed sinus tachycardia and arrhythmias were not noted. He had normal echocardiogram findings with no features suggesting myocarditis.

Based on the clinical and investigation findings, diagnosis of Guillain–Barré syndrome was reached and intravenous immunoglobulin 0.4 g/kg/day was administered for five days. Patient showed good clinical response to treatment and the muscle power improved to 4/5 in all muscle groups during the ward stay, though the patient remained areflexic. His symptoms of Raynaud’s phenomenon, palpitations and postural giddiness showed dramatic improvement during the ward stay and on discharge patient was free of symptoms. His pulse rate had reduced to 80 beats per minute and blood pressure was 120/80 mmHg, both supine and erect. He was followed up to six months and had completely recovered with normal muscle power with 1+ reflexes, and had not experienced Raynaud’s phenomenon even with exposure to cold weather.

## Discussion

Guillain–Barré syndrome is an immune mediated disease, manifesting as an acute polyradiculo- neuropathy involving the peripheral nervous system. Clinical manifestations are preceded by an antecedent event in two thirds of the patients [1],[2], commonly an upper respiratory tract infection or a diarrhoeal illness. Pathogenesis involves production of auto antibodies against the infectious agent that cross react against gangliosides and glycolipids (GM1 and GD1b) that are distributed along the myelin sheaths of the peripheral nervous system. This causes defective propagation of electrical impulses thus resulting in marked delay or absence of electrical impulse along the nerve fibers resulting in flaccid paralysis. There are several sub-types of Guillain–Barré syndrome identified depending on clinical presentation, involvement of the particular nerves and pathology; (1) Acute inflammatory demyelinating poluneuropathy, (2) Acute motor axonal neuropathy, (3) Acute motor sensory axonal neuropathy, (4) Miller Fisher syndrome, (5) Acute panautonomic neuropathy and (6) Pure sensory Guillain–Barré syndrome.

Patients with Guillain–Barré syndrome commonly present with acute or sub-acute symmetrical weakness of lower limbs with ascending paralysis extending to upper limbs, neck muscles, bulbar muscles, cranial nerves and respiratory muscles needing ventilatory support [2],[7],[8]. Muscle involvement is proximal more than the distal muscle groups and commonly with diminished or absent deep tendon reflexes. It is not uncommon to notice mild paresthesia or pain by the patients.

Autonomic dysfunction is also seen in 60% of the patients with Guillain–Barré syndrome [3]. Rarely it can be the presenting manifestation of Guillain–Barré syndrome [2],[4],[5]. Clinical features of dysautonomia commonly seen in relation to cardiovascular system are sinus tachycardia, sinus bradycardia, other cardiac arrhythmias (both tachy and bradyarrythmias), hypertension, postural hypotension and wide fluctuations of pulse and blood pressure. Tonic pupils, hyper salivation, anhydrosis or excessive sweating, urinary sphincter disturbances, constipation and gastric dysmotility are recognized as a part of autonomic dysfunction [3],[9].

Raynaud’s phenomenon is the triad of pallor, cyanosis and redness of the acral regions of the body (hands and feet) due to episodic digital vasospasms. Classically it is brought about by cold environment or emotions [10]. Pallor is a result of ischaemia due to vasospasms, cyanosis due to deoxygenation of blood and redness being the result of reperfusion following reversal of the spasms. Two subtypes are described, primary Raynaud’s which is not associated with any other illness and secondary Raynaud’s phenomenon, where vasospasms are associated with another disease, commonly autoimmune diseases, mixed connective tissue disease and scleroderma. Pathogenesis of primary Raynaud’s phenomenon is not fully understood but vascular, intravascular and neural mechanisms are proposed [6],[11]. Of the neural mechanisms, central impairment of the autonomic function resulting in Raynaud’s phenomenon is suggested by the research done by Koszewicz et al. [12]. Mallipeddi et al. concluded that primary Raynaud’s phenomenon is associated with sympathetic denervation in chronic autonomic failure [10]. Sympathetic over-activity provoking vasospasm by exaggerated vascular response is also suggested as one of the mechanisms causing Raynaud’s phenomenon [11],[13]. Hyperactivity of the α2c adrenorecepters is also postulated as one of the mechanisms [6]. But experiments done by Fagius et al. showed neither hypersensitivity of the vessels to sympathetic bursts nor abnormal increase in sympathetic outflow was present in patients with Raynaud’s phenomenon [14]. Further studies are required for a better knowledge regarding the contribution of sympathetic nervous system or dysautonomia in Raynaud’s phenomenon.

Although several etiologies are described in-relation to Raynaud’s phenomenon as primary Raynaud’s’s disease, occupational, haematological, autoimmune, connective tissue diseases, vasculitis, thrombo-embolic disease, carpal tunnel syndrome, thoracic outlet syndrome, reflex sympathetic dystrophy, pheochromocytoma, acromegaly, lung adenocarcinoma and Fabry s disease, extensive literature survey did not reveal Raynaud’s phenomenon in association with Guillain–Barré syndrome. Common pathological features in both conditions are dysautonomia with altered sympathetic activation.

The patient in this case report presented with Raynaud’s phenomenon and subsequently developed features of Guillain–Barré syndrome with further supportive evidence from CSF and nerve conduction studies. He also had other features of autonomic dysfunction as postural hypotension. Weakness and neurological symptoms resolved following treatment for Guillain–Barré syndrome and Raynaud’s phenomenon also resolved permanently.

## Conclusion

We report the first case of Guillain–Barré syndrome presenting with Raynaud’s phenomenon, which highlights the importance of considering the possibility of Guillain–Barré syndrome in a patient presenting with de novo Raynaud’s phenomenon. More classic features of Guillain-Barre syndrome may follow soon after. This is important for close observation and prompt treatment. The exact pathophysiological mechanisms linking above two entities can be postulated as dysautonomia but need further evaluation with experimental studies.

### Consent

Written informed consent was obtained from the patient for publication of this case report. A copy of the written consent is available for review by the Editor-in-chief of this journal.

## Abbreviations

AIDP: Acute inflammatory demyelinating polyneuropathy

ANCA: Anti-neutrophil cytoplasmic antibodies

CSF: Cerebrospinal fluid

HIV: Human immunodeficiency virus

## Competing interests

The authors declare that they have no competing interests.

## Authors’ contributions

HW made the clinical diagnosis and supervised the manuscript drafting. SSCG drafted the first manuscript, reviewed the literature and involved in direct management of the patient. Both authors read and approved the final manuscript.

## Authors’ information

HW (MBBS, MD, FRCP (Edin), FRCP (Lond), FCCP ) is a Consultant Physician working at Teaching Hospital, Kandy, Sri Lanka. SSCG (MBBS) is a Registrar in Medicine attached to the Teaching Hospital, Kandy, Sri Lanka.

## References

[B1] Winer JB Hughes RA Anderson MJ Jones DM Kangro H Watkins RP A prospective study of acute idiopathic neuropathy. II Antecedent events J Neurol Neurosurg Psychiatry 1988 51 613 618 10.1136/jnnp.51.5.613 3404161 PMC1033063

[B2] Seneviratne U Guillain-Barré syndrome Postgrad Med J 2000 76 774 782 10.1136/pmj.76.902.774 11085768 PMC1741839

[B3] Patel MB Goyal SK Punnam SR Pandya K Khetarpal V Thakur RK Guillain-Barré syndrome with asystole requiring permanent pacemaker: a case report J Med Case Rep 2009 3 5 10.1186/1752-1947-3-5 19126210 PMC2628935

[B4] Ferraro-Herrera AS Kern HB Nagler W Autonomic dysfunction as the presenting feature of Guillain-Barré syndrome Arch Phys Med Rehabil 1997 78 7 777 779 10.1016/S0003-9993(97)90089-7 9228884

[B5] Cortelli P Contin M Lugaresi A Baruzzi A Montagna P Severe dysautonomic onset of Guillain-Barré syndrome with good recovery. A clinical and autonomic follow-up study [abstract] Ital J Neurol Sci 1990 11 2 159 162 10.1007/BF02335559 2361852

[B6] Herrick AL Pathogenesis of Raynaud’s phenomenon Rheumatology 2005 44 5 587 596 10.1093/rheumatology/keh552 15741200

[B7] Flachenecker P Autonomic dysfunction in Guillain-Barré syndrome and multiple sclerosis J Neurol 2007 254 2 96 101 10.1007/s00415-007-2024-3 17503142

[B8] Pikula JR Guillain-Barre syndrome: a case report J Can Chiropr Assoc 1995 39 2 80 83

[B9] Zochodne DW Autonomic involvement in Guillain-Barré syndrome: a review Muscle Nerve 1994 17 10 1145 1155 10.1002/mus.880171004 7935521

[B10] Mallipeddi R Mathias CJ Raynaud’s phenomenon after sympathetic denervation in patients with primary autonomic failure: questionnaire survey BMJ 1998 316 438 10.1136/bmj.316.7129.438 9492669 PMC2665585

[B11] Coffman JD Pathogenesis and treatment of Raynaud’s phenomenon Cardiovasc Drugs Ther 1990 4 1 45 51 10.1007/BF00053426 2285650

[B12] Koszewicz M Gosk-Bierska I Bilińska M Podemski R Budrewicz S Adamiec R Slotwinski K Autonomic dysfunction in primary Raynaud’s phenomenon Int Angiol 2009 28 2 127 131 19367242

[B13] Urbano FL Raynaud’s phenomenon Hosp Physician 2001 ᅟ 27 30 http://www.turner-white.com/pdf/hp_sep01_raynaud.pdf http://www.turner-white.com/pdf/hp_sep01_raynaud.pdf

[B14] Fagius J Blumberg H Sympathetic outflow to the hand in patients with Raynaud’s phenomenon Cardiovasc Res 1985 19 5 249 253 10.1093/cvr/19.5.249 3995520

